# Repeatability of biometric measures from the LenStar LS900 in a cataractous population

**DOI:** 10.1371/journal.pone.0321786

**Published:** 2025-05-05

**Authors:** Achim Langenbucher, Nóra Szentmáry, Jascha Wendelstein, Alan Cayless, Peter Hoffmann, David Cooke

**Affiliations:** 1 Department of Experimental Ophthalmology, Saarland University, Homburg, Germany; 2 Dr. Rolf M. Schwiete Center for Limbal Stem Cell and Aniridia Research, Saarland University, Homburg, Germany; 3 Department of Ophthalmology, Semmelweis-University, Budapest, Hungary; 4 Department of Ophthalmology, Ludwig-Maximilians University, Munich, Germany; 5 School of Physical Sciences, The Open University, Milton Keynes, United Kingdom; 6 Augen- und Laserklinik Castrop-Rauxel, Castrop-Rauxel, Germany; 7 Great Lakes Eye Care, Saint Joseph, Michigan, United States of America; 8 Department of Neurology and Ophthalmology, Michigan State University, College of Osteopathic Medicine, East Lansing, Michigan, United States of America; Sanmenxia Central Hospital, Henan University of Science and Technilogy, CHINA

## Abstract

**Purpose:**

To investigate the repeatability of biometric measures and assess interactions between their uncertainties for use in an error propagation model, using patient data.

**Methods:**

Cross-sectional non-randomised study evaluating a dataset containing 969 LenStar 900 biometric measurements taken before cataract surgery. Only complete scans with at least 3 successful measurements for each eye performed on the same day were considered. For each sequence, the aggregated mean (AMEAN) and population standard deviations (ASD) were derived. The within-subject standard deviation Sw was extracted for: corneal thickness, CCT, anterior chamber depth ACD, lens thickness LT, axial length AL, corneal diameter WTW, and the keratometric power vector components equivalent power K_EQ_, and the projections of corneal astigmatism K_C0_ and K_C45_. Correlations between the uncertainties were assessed using Spearman rank correlations.

**Results:**

For the 266 eyes matching the inclusion criteria, Sw was 3.6/ 24.7/35.5/ 17.7/ 107.5 µm for CCT/ ACD/ LT/ AL WTW and 0.18/ 0.12/ 0.10 dioptres for KEQ/ KC0/ KC45. The keratometric axis ASD is inversely proportional to the keratometric astigmatism AMEAN. LT and ACD uncertainties are strongly negatively correlated, with K_EQ_ and K_C0_ uncertainties moderately correlated.

**Conclusions:**

The uncertainty and correlation data presented here could be used to define a Monte-Carlo based error propagation model mapping the biometric measures and uncertainties to variations in predicted refraction after cataract surgery. We recommend using power vector components for error propagation models since the large decay over keratometric astigmatism makes keratometric axis uncertainty unreliable.

## Background

Intraocular lens (IOL) power calculation is still a challenge in cataract surgery, especially in eyes with uncommon biometric measures or unusual combinations of biometric parameters. There are several sources of prediction errors for the resulting postoperative spectacle refraction: firstly, we have measurement errors in all of the biometric values used in the lens power calculation strategy. Secondly, we have formula errors resulting mostly from simplifications in the pseudophakic optical models or assumptions which might be invalid. Thirdly, we have labelling errors of the IOL itself. And finally, there might be deterministic changes in the eye which are not considered in our calculation concept, or stochastic changes which could not be predicted [1–5].

For calculation of stigmatic lenses the main factors that could affect the prediction are: the uncertainties in the axial length (AL), the mean corneal radius R or mean keratometric power (K), and supplemental measures such as central corneal thickness (CCT), anterior chamber depth (ACD, measured from corneal epithelium to the front apex of the crystalline lens) and the central thickness of the crystalline lens (LT). If the calculation concept considers the cornea as a ‘thick lens’, then the uncertainties of the corneal back surface curvature may also affect the results.

However, when calculating toric lenses the overall uncertainties in R and K alone are not sufficient. The corneal radius or keratometric power could show uncertainties in both cardinal meridians and in the orientation of the flat/steep axis, making the situation more complex [6–8]. Similar problems occur in relation to the labelling error of the IOL: With a stigmatic lens only the uncertainty in the IOL equivalent power is required, but with toric IOL the uncertainties in the equivalent and toric power as well as any misalignment of the toric axis must be considered [2].

The variability of biometric measures from a number of modern optical biometers (including the IOLMaster 700, LenStar 900, Pentacam AXL, OA-2000, and Galilei G6) has been assessed in a number of previous studies [4,7,9–23]. These studies typically employ a measurement protocol with at least three repeats of each measurement, in order to assess either the inter-operator or intra-operator variability, in terms of the within-subject standard deviation (Sw) and other statistical measures [6]. What has not previously been fully explored is the interactions or correlations between the various biometric measures and between their uncertainties. Also, any trends in the variances of each parameter across the measurement range (heteroscedasticity) are typically not considered [1,2,8]. It would also be reasonable to expect that any correlations between individual biometric measures would be reflected in correlations between the uncertainties. If this were to be the case, it would rule out the use of simple Gaussian error propagation models [8].

In contrast, modern Monte-Carlo based error propagation models have the advantage of being able easily to include information on the distribution of the uncertainties in the biometric measures (e.g., uniformly distributed instead of being normally distributed) or correlations between the uncertainties (with a correlation matrix or variance-covariance matrix) as well as any trend of the uncertainties over the parameter range [1,2].

The **purpose of the present study** was to analyse a dataset of measurements from the LenStar optical biometer including multiple measurements of eyes before cataract surgery

- To evaluate the within-subject standard deviation Sw for all relevant biometric measures as an overall metric describing the variation of data for multiple measurements,
- To extract the trend of all biometric parameter uncertainties over the entire parameter range as a measure for heteroscedasticity, and
- To analyse all correlations between the measures themselves and between the measure uncertainties to be used in an advanced error propagation model to predict the spectacle refraction error after cataract surgery with implantation of stigmatic or toric intraocular lenses.

## Methods

### Dataset for our evaluation

The dataset used in this study contained biometric measurements performed at the Great Lakes Eye Care Center (St. Joseph, Michigan, USA) with the LenStar 900 (Haag-Streit, Köniz, Switzerland) between March 2019 and March 2023. This raw dataset contained a total of N = 3144 records. All procedures carried out in studies involving human participants were in accordance with the ethical standards of the Ärztekammer des Saarlandes and with the 1964 Helsinki declaration and its later amendments or comparable ethical standards. The local ethics committee (IRB) has provided a waiver for this study (Ärztekammer des Saarlandes, 157/21), as all data processed in this study were already anonymized at source before being transferred to us for processing. This precludes any back-tracing of the identity, and therefore informed consent of the patients was not necessary.

This article does not contain any studies on animals performed by any of the authors.

The anonymized data were exported and transferred to us as an Excel (.xlsx) data file. The following parameters were included in the exported data: patient ID, date of birth, examination date, sex (female or male), the laterality (left or right eye), flat (K1) and steep (K2) keratometric power, both in diopters (D), together with the flat axis Axis1 in degrees, axial length (AL) in mm, central corneal thickness (CCT) in mm, aqueous depth (AD) in mm (measured from corneal endothelium to the lens front apex), central thickness of the crystalline lens (LT) in mm, horizontal corneal diameter (WTW) in mm, pupil size (PUP), the pupil barycentre defined as the chord between the coaxially sighted light reflex and the pupil centre in Cartesian coordinates (ICX horizontally in mm, ICY vertically in mm), and the iris barycentre defined as the chord between the limbal centre and the coaxially sighted light reflex in Cartesian coordinates (PCX horizontally in mm, PCY vertically in mm) [24,25].

In order to exclude potentially correlated data due to similarities or symmetries between both eyes of an individual [25–27] only one eye from each subject was included in this study. Where measurements of both eyes were available, one eye was randomly selected for inclusion in this study. Subjects with missing data were excluded. Only records containing a sequence of at least 3 complete measurements were selected. The data sent to us for processing on May 14 2024 were transferred to Matlab (Matlab 2022b, MathWorks, Natick, USA) for further processing.

### Data pre-processing in Matlab

From the corneal front surface data (K1, K2, Axis1) and the keratometer index used in the measurement (nK 1.3375), we extracted the corneal radii in the flat meridian R1 = 1000·(nK-1)/K1 and steep meridian R2 = 1000·(nK-1)/K2 both in mm, and the mean corneal radius R, calculated as the harmonic mean of R1 and R2 (R = 2·R1·R2/(R1+R2)) [1,2]. Keratometric astigmatism was derived as K_AST_= K2-K1. The standard notation of keratometry (K1, K2, Axis1) was converted to 3D power vector components with equivalent power K_EQ_ = 0.5·(K1+K2) and the 2 astigmatic power vector components in terms of projections of keratometric astigmatism to the 0°/90° axis (K_C0_ = ½·K_AST_·cos(2·Axis1)) and the 45°/135° axis (K_C45_ = ½·KAST·sin(2·Axis1)). The anterior chamber depth (ACD), one of the most commonly used parameters for IOL power calculation, was derived from CCT and AD as ACD = CCT+AD. The age of each patient was calculated as the difference between the date of birth and the date of the examination.

### Data processing in Matlab and statistics

For each eye, the values of each parameter from the measurement sequence were aggregated into a corresponding arithmetic mean value (.)_AMEAN_ and (population) standard deviation (.)_ASD_. For the keratometric axis measurements we performed a periodic correction for the calculation of the mean and the standard deviation. The overall within-subject standard deviation Sw for the dataset was calculated for each parameter as $s_{w}=\sqrt{\frac{1}{N}\underset{i}{\sum }{\left(.\right)}_{ASD}^{2}}$. To assess heteroscedasticity, the (.)_ASD_ was analysed as a function of the ()_AMEAN_ values. To investigate interactions between the uncertainties of the biometric measures, the differences of all measurements and their (.)_AMEAN_ values were cross-correlated (Spearman correlation coefficient ρ and significance level p based on a first order error of α = 5%) for all parameters. In addition, to support the development of advanced error propagation models, we calculated the variance-covariance matrix for the differences of all measurements and their (.)_AMEAN_ values [6].

The main statistical parameters including the arithmetic mean, the SD, the median, and the lower and upper boundary of the 95% confidence interval (i.e., to the 2.5% and 97.5% quantiles) for the most relevant (.)_AMEAN_ and (.)_ASD_ values are summarised in tables. Correlation plots are shown for the interaction of the biometric parameters themselves and for the interactions of the parameter uncertainties. Negative correlations are marked in blue colours and positive correlations in red colours. Correlations with a correlation coefficient |ρ|>= 0.6 were considered as strong. Scatterplots were used to show the trend of the (.)_ASDi_ values as a function of the (.)AMEAN values (together with least squares linear fit lines).

Double angle plots with a 95% error ellipse were used to display the differences of all measurements and their (.)_AMEAN_ values of the 2 astigmatic power vector components in the measurement sequence from the centroid. In addition, a 95% confidence ellipsoid was derived. This shows the differences of all measurements and their (.)_AMEAN_ values of the 3D power vector components from the 3D centroid.

## Results

Following the application of the selection criteria, a total of N = 969 records of measurements from 266 eyes (127 right/ 139 left) were selected for inclusion in the analysis from the original database of 3144 biometric measurements. The records for 174/ 51/ 20/ 10/11 eyes included 3/ 4/ 5/ 6/ 7–10 measurements per eye respectively. Table 1 displays explorative data for the aggregated mean and standard deviation values of the most relevant biometric parameters. The within-subject standard deviations were: Sw = 0.0034 mm for CCT, 0.0247 mm for AD and ACD, 0.0355 mm for LT, 0.0177 mm for AL, 0.1167 D for K1, 0.1766 D for K2, 0.1823 D for K_AST_, 0.1187 D for K_EQ_, 0.0962 D for K_C0_, 0.0716 D for K_C45_, and 0.1076 mm for WTW. The Sw values for the pupil size, ICX, ICY, PCX and PCY were 0.3016 mm/ 0.0672 mm/ 0.0967 mm/ 0.0369 mm and 0.0482 mm respectively.

**Table 1:** Explorative data of the most relevant aggregated biometric measures in terms of mean value (.)_AMEAN_ and population standard deviation (.)_ASD_ of the sequence of repeat measurements for each eye. CCT refers to the central corneal thickness, ACD to the anterior chamber depth measured from the corneal epithelium to the lens front apex, LT to the central thickness of the crystalline lens, AL to the axial length, K1 and K2 to the keratometric powers in the flat and steep meridians, KAST to the keratometric astigmatism, and K_EQ_, K_C0_ and K_C45_ to the 3 power vector components comprising equivalent power and projection of the keratometric astigmatism to the 0°/90° and 45°/135° meridian. Please note that all (.)_ASD_ values are scaled by x100.

N = 249		CCT in mm	ACD in mm	LT in mm	AL in mm	K1 in D	K2 in D	K_AST_ in D	K_EQ_ in D	K_C0_ in D	K_C45_ in D
Aggregated (.)_AMEAN_	Mean	0.5413	3.3276	4.4720	24.5091	43.7509	44.8944	1.1435	44.3227	0.3190	0.0043
	Standard deviation	0.0363	0.4094	0.4214	1.6121	1.5716	1.6332	0.8115	1.5505	0.5464	0.2930
	Median	0.5419	0.3436	4.5118	24.5716	43.8119	44.9313	0.9779	44.3560	0.3235	0.0082
	2.5% quantile	0.4668	2.5663	3.5253	21.6219	40.7375	41.9593	0.1695	41.3581	-0.8822	-0.6170
	97.5% quantile	0.6166	4.0604	5.1994	27.4183	46.4333	47.9241	3.3471	47.0771	1.4550	0.5830
Aggregated (.)_ASD_ x100	Mean	0.2810	1.7528	2.3022	1.3518	9.6456	14.5412	15.2439	9.8935	8.2392	8.9595
	Standard deviation	0.1876	1.7394	2.7129	1.1473	6.5881	10.0329	10.0160	6.5729	4.9804	3.9831
	Median	0.2544	1.2193	1.2226	1.0241	8.0219	11.8150	13.0800	8.8714	7.4772	5.2413
	2.5% quantile	0.0406	0.1640	0.1719	0.2450	1.5944	1.6285	2.4294	1.2960	1.4122	1.1506
	97.5% quantile	0.8535	7.1872	11.2735	3.8255	26.7732	39.5197	39.6565	27.2107	19.9600	17.8649

**Fig 1:**
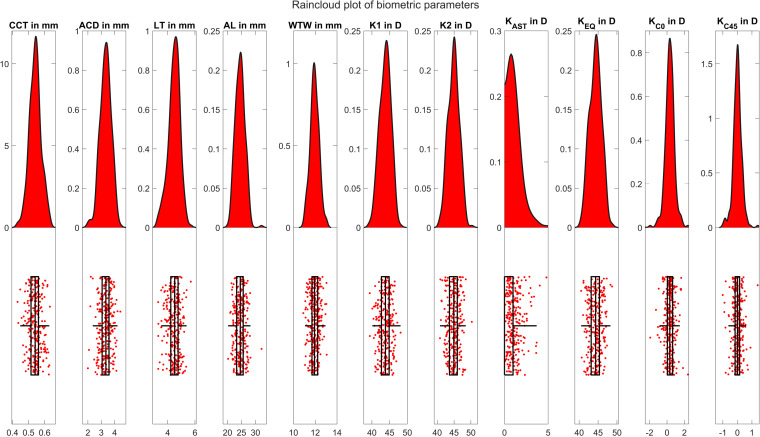
Raincloud plot showing the distributions of the aggregated mean values (.)_AMEAN_ for the most relevant biometric measures. From the graph it can be seen that most of the distributions are not represented by a Gaussian normal distribution. CCT, ACD, LT, AL and WTW refer to the central corneal thickness, anterior chamber depth, central thickness of the crystalline lens and axial length respectively, and K1, K2, K_AST_, K_EQ_, K_C0_ and K_C45_ to the keratometric powers in the flat and steep meridians, keratometric astigmatism, and the 3 power vector components of keratometry comprising the equivalent power and projections of the astigmatism to the 0°/90°° and 45°/135° meridian.

The raincloud plot in Fig 1 displays the distributions of the aggregated mean values (.)_AMEAN_ for the most relevant biometric measures. From the graph it can be seen that most of these biometric measures are not fully represented by a normal distribution.

**Fig 2:**
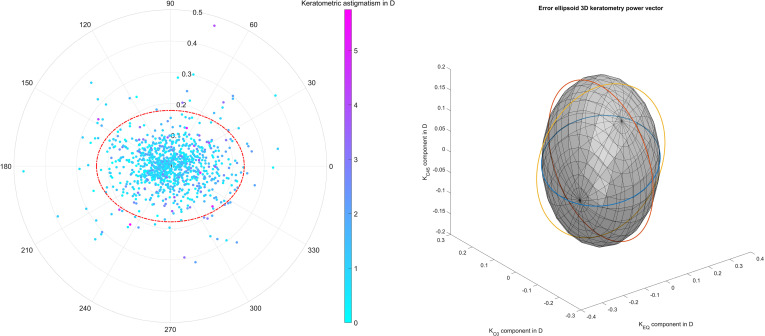
The left-hand graph is a double angle plot showing the variation of the keratometric data in the measurement sequence for each eye for the two astigmatic power vector components. The red dash dotted line indicates the 95% error ellipse derived from the variance-covariance matrix. The data points are colour coded according to the keratometric astigmatism K_AST_ value as indicated in the colour bar. The graph on the right side shows the 95% error ellipsoid derived from the 3D keratometric power vector (equivalent power K_EQ_, and the 2 astigmatic power vector components K_C0_ and K_C45_ as projections of the keratometric astigmatism to the 0°/90° and the 45°/135 meridian). The 3 projections of the 2D 95% error ellipses are indicated as blue, red, and yellow lines.

The graph on the left of Fig 2 is a double angle plot showing the variation of the keratometric data in the measurement sequence for each eye for the two astigmatic power vector components. From this plot it can be seen that the uncertainties of the 2 astigmatic power vector components are somewhat uncorrelated and are not strictly dependent on the amount of keratometric astigmatism K_AST_. The red dash-dotted line indicates the 95% error ellipse derived from the variance-covariance matrix. The data points are colour coded according to the keratometric astigmatism K_AST_ value as indicated in the colour bar. The graph on the right side shows the 95% error ellipsoid derived from the 3D keratometric power vector based on the variance-covariance matrix. The 3 projections of the 2D 95% error ellipses are added to the plot as blue, red and yellow lines.

**Fig 3:**
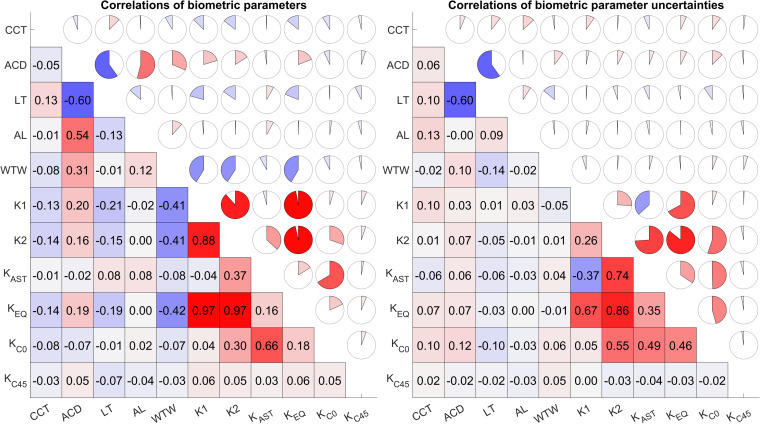
Correlation plots showing the Spearman rank correlation coefficients ρ for the most relevant biometric parameters (left graph) and the uncertainties of the most relevant parameters (right graph). CCT, ACD, LT, AL and WTW refer to the central corneal thickness, anterior chamber depth, central thickness of the crystalline lens and axial length respectively, and K1, K2, K_AST_, K_EQ_, K_C0_ and K_C45_ to the keratometric powers in the flat and steep meridians, keratometric astigmatism, and the 3 power vector components of keratometry comprising equivalent power and projections of the astigmatism to the 0°/90°° and 45°/135° meridian. The correlation coefficient is colour-coded in red for positive and in blue for negative correlations. Strong positive correlations are shown in progressively darker shades of red, and negative correlations in progressively darker shades of blue. Lighter shades indicate weaker correlations in each direction, with light grey/ white cells indicating the weakest or zero correlations.

The left graph of Fig 3 displays the correlation coefficients ρ between the most relevant biometric parameters and the right graph shows the correlations between the measurement uncertainties. From the left graph it can be seen that there is a strong negative correlation between LT and ACD, and strong positive correlations between K1 and K2, KEQ and K1/ K2, and K_C0_ and K_AST_. The measurement uncertainties shown on the right graph again show a strong negative correlation between LT and ACD and strong positive correlations between K1 and K_EQ_, K2 and K_EQ_/ K_AST_.

Table 2 lists the scaled (x1000) variance-covariance matrix of the biometric measure uncertainties to be used in an advanced error propagation model. On the diagonal the variances are provided (in bold), and in the off-diagonal elements the covariances are shown.

**Table 2:** Variance-covariance matrix of the biometric measure uncertainties to be used for an advanced error propagation model. The variances are shown (in bold) on the diagonal, and the covariances are shown in the off-diagonal elements. All values are scaled by x1000. CCT, ACD, LT and WTW refer to the central corneal thickness, anterior chamber depth, central thickness of the crystalline lens, axial length, and horizontal corneal diameter respectively. K1, K2, K_AST_, K_EQ_, K_C0_ and K_C45_ refer to the keratometric powers in the flat and steep corneal meridians, the corneal astigmatism, and to the 3 keratometric power vector components with the equivalent power K_EQ_ and the 2 astigmatic components as projections of the astigmatism to the 0°/45° meridian K_C0_ and the 45°/135° meridian K_C45_.

X1000	CCT	ACD	LT	AL	WTW	K1	K2	K_AST_	K_EQ_	K_C0_	K_C45_
**CCT**	**0.0117**										
ACD	0.0028	**0.7082**									
LT	0.0183	−0.7191	**1.5015**								
AL	0.0081	−0.0335	0.1157	**0.3485**							
WTW	−0.0110	0.2163	−0.3223	0.0599	**12.0277**						
K1	0.0503	-0.0735	0.3516	0.0448	−0.6694	**14.2708**					
K2	−0.0130	0.1561	−0.2687	−0.0484	0.1419	6.1057	**31.9104**				
K_AST_	−0.0633	0.2296	−0.6203	−0.0932	0.8113	−8.1651	25.8047	**33.9698**			
K_EQ_	0.0186	0.0413	0.0414	−0.0018	−0.2637	10.1883	19.0081	8.8198	**14.5982**		
K_C0_	0.0294	0.2786	−0.3762	−0.0229	0.6183	0.9521	10.5910	9.6389	5.7715	**9.3030**	
K_C45_	0.0148	−0.0932	0.1013	−0.0136	0.1309	−0.2064	−0.6701	−0.4637	−0.4382	−0.0050	**5.2933**

**Fig 4:**
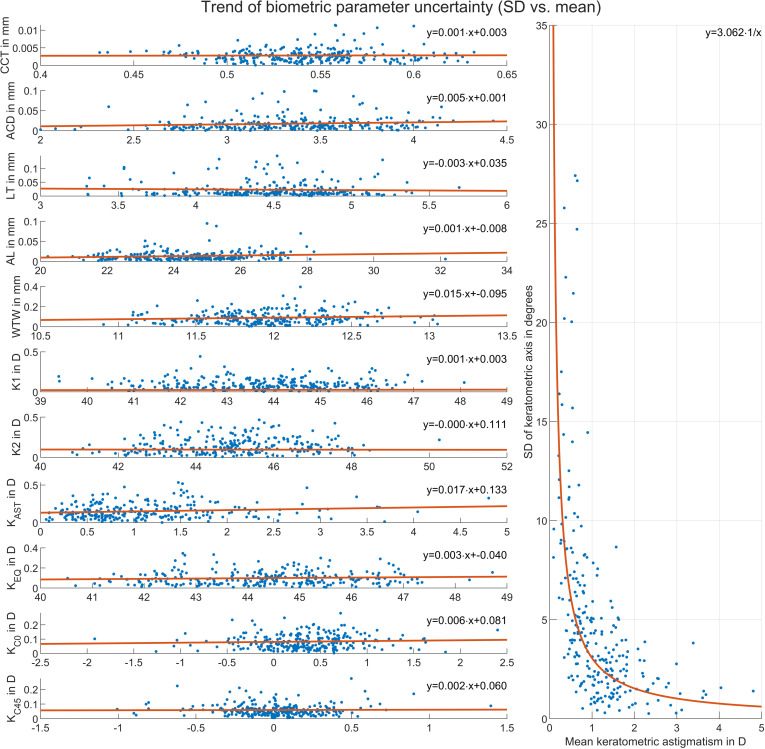
Trend of the most relevant biometric parameter uncertainties as a function of their aggregated mean values (.)_AMEAN_ (left graph) and uncertainty of the keratometer axis (periodically corrected) as a function of the aggregated mean keratometric astigmatism (K_AST_)_AMEAN_ (right graph). CCT, ACD, LT, AL and WTW refer to the central corneal thickness, anterior chamber depth, central thickness of the crystalline lens and axial length respectively, and K1, K2, K_AST_, K_EQ_, K_C0_ and K_C45_ to the keratometric powers in the flat and steep meridians, keratometric astigmatism, and the 3 power vector components of keratometry comprising equivalent power and projections of the astigmatism to the 0°/90°° and 45°/135° meridian. For all plots the trend line in terms of a least squares fit is shown with parameters given in the legends. From the left plots it can be seen that some of the uncertainties show a slight positive or negative trend (linear fit line with positive or negative slope) which refers to heteroscedasticity of the parameter variation. The uncertainty in the keratometer axis (right graph) strictly follows an inverse proportionality according to **y** ~ 1/x. This means that for low or moderate corneal astigmatism the variation of the astigmatism axis in repeat measurements is significantly larger than for moderate or high values of corneal astigmatism. In clinical practice, this implies that providing one single value for the astigmatism axis uncertainty might be insufficient. Instead, we should provide the corresponding metrics for the power vector components as the measurement uncertainty for these power vector components appears to be mostly unaffected by the amount of astigmatism.

Fig 4 provides some insight into the trend of the most relevant biometric parameter uncertainties as a function of their aggregated mean values (.)_AMEAN_ (left graph) and into the uncertainty of the keratometer axis as a function of the aggregated mean keratometric astigmatism (K_AST_)_AMEAN_ (right graph). All plots include a trend line in terms of a least squares fit with the parameters of each trend line given in the legend of each subplot. The plots on the left indicate that for most variables the uncertainty shows some slight increase (positive slope of the trend line) or decrease (negative slope of the trend line) with increase of the aggregated mean value. The uncertainty in the keratometer axis as a function of keratometric astigmatism (right graph) strictly follows an inverse proportionality according to y ~ 1/x.

## Discussion

Optical biometry today is widely regarded as the gold standard in ocular biometry. Compared to ultrasound, optical biometry provides keratometry measurements and with some devices also corneal topography or tomography in addition to the standard measurements of relevant distances in the eye. The precision is much higher compared to ultrasound, and the non-contact measurement is performed within seconds [4,6,8,10–23,28–32].

Driven by improvements in ocular biometry, lens power calculation concepts have been upgraded in the last 2 decades to include more and more measures of the eye, in order to improve the lens power prediction or the prediction of the postoperative refractive outcome. At the same time, increasing numbers of new lens types in the premium lens segment such as ‘enhanced depth of focus’ lenses or ‘monofocal plus’ lenses have been launched. These more advanced designs require correspondingly more reliable prediction of the refraction after cataract surgery.

However, all biometric measures show variations with repeated measurements, either in the same session or in a subsequent session carried out by either the same examiner or a different examiner [9–19]. This means that ocular biometry is a simple ‘snapshot’. Any variations of measurements cause a corresponding variation in the refraction predicted by any lens power formula or raytracing [1,2,8]. The classical way of understanding the effect of variation of the refractive outcome resulting from such uncertainties in the biometric measure is to set up a Gaussian error propagation model. This requires knowledge of all of the variances of the biometric measures and the gradient of the target function (postoperative refraction) on the effect predictors (biometric measures). However, such simplified error propagation models do not take into account any crosstalk between any of the measured parameters or between measurement uncertainties and furthermore these models assume that all measurement errors are normally distributed [8].

Today, the larger capacities of personal computers allow more powerful Monte-Carlo error propagation models to be implemented instead of Gaussian error propagation models. These can easily deal with any distribution of the biometric parameters, and any distribution and correlation of the measurement uncertainties [1,2]. A Monte-Carlo simulation requires a large number of sweeps, and this number increases with the complexity of the mapping function from the biometric measures to the target parameter (e.g., the nonlinearity), the number of predictors and their correlations. Such an advanced error propagation model requires either the variances of all predictors and the correlation matrix, or the variance-covariance matrix containing both the variances and the correlations. In addition, we must determine whether the biometric measure uncertainties are constant over the entire parameter range or whether they show some trend in terms of heteroscedasticity [6].

The aim of the present study was, by generating the necessary data incorporating all relevant parameters from a modern optical biometer, to create the foundations of an advanced error propagation model for estimating the variation in the predicted IOL power or the variation in the predicted refraction after cataract surgery. The heteroscedasticity of the biometric measures was assessed by monitoring the trends of the measure uncertainties over the entire parameter range, and in order to account for correlations between biometric measures and measure uncertainties a variance-covariance matrix was calculated. The main results of our study are that the axial distances show an excellent repeatability with within-subject standard deviations of Sw = 3 µm for CCT, 25 µm for AD and ACD, 36 µm for LT, and 18 µm for AL, based on at least 3 repeat measurements per eye. In contrast, Sw of WTW appears much worse at 108 µm, probably due to a lack of a high contrast ‘sharp edge’ between the cornea and the sclera. These results match those of other studies on the LenStar 900 [12,14,16,19]. The repeatability of the keratometry seems to be much worse with Sw values of 0.12 D and 0.18 D for K1 and K2 respectively, 0.18 D for K_AST_, and 0.12 D/ 0.10 D/ 0.07 D for the 3 keratometric power vector components comprising equivalent power and the projections of keratometric astigmatism to the 0°/90° and 45°/135° meridian [29]. However, the most important results are confirmation that the standard notation for keratometry is not sufficient for any error propagation model and that the crosstalk between biometric measures and their uncertainties must also be considered. The standard notation for keratometry in terms of sphere, astigmatism and axis or with power in the flat and steep meridian and axis has the drawback that the uncertainty of the keratometer axis is strictly dependent on the amount of keratometric astigmatism as shown in the right graph of Fig 4. This means that using any uncertainty metrics for the keratometric axis such as Sw for the entire astigmatism range will not work because the axis uncertainty follows a trend line of 1/K_AST_: for low astigmatism values the variation in the keratometer axis is extremely high ((Axis1)_ASD_ up to 30°), whereas for larger astigmatism repeatability is much better [6]. Additionally, using the uncertainty metrics for the flat or steep corneal meridian K1 and K2 overlooks any potential variation in the keratometer axis in the sequence of measurements. Therefore, we decided not to provide Sw data for the keratometric axis since this value depends mostly on the dataset and the portion of small or larger astigmatism values. Instead of the standard notation, the component notation should be used as this exhibits no visible trend of the uncertainty over the parameter range for all 3 keratometric power vector components and even for the 2 astigmatic power vector components as shown on the left graph of Fig 4. From Fig 3 it can be seen that the biometric parameters used for IOL power calculation and also the parameter uncertainties are not uncorrelated. If we consider moderate/ strong correlations as those with a correlation coefficient larger than or equal to 0.3/ 0.6 and if, as recommended here, we use the vector component notation of keratometry (K_EQ_ for stigmatic IOLs and K_EQ_/ K_C0_/ K_C45_ for toric IOLs) instead of the standard notation, we find a strong negative correlation between LT and ACD, a moderate correlation of ACD with AL and WTW, and no significant correlation within the keratometric power vector components or between the power vector components and other biometric measures. For the biometric measure uncertainties which have to be considered in the error propagation model using the correlation matrix or the variance-covariance matrix, we again see a strong negative correlation between LT and ACD and a moderate correlation between K_EQ_ and K_C0_ [1,2,6].

In a previous study [6] we investigated measurement uncertainties with the IOLMaster 700 based on a population before cataract surgery based on repeat measurements. In addition to distances in the eye such as AL, CCT, ACD, LT or WTW we evaluated the uncertainties of corneal front surface power (keratometry) and back surface power, and the TK values (total keratometry) to provide normative data for built-up of a classical error propagation model. We also derived the correlations between the measurement uncertainties required to establish a more advanced error propagation model which would also take account of the crosstalk between measurement uncertainties. However, this previous paper did not address the issue of the inverse proportionality between the uncertainty in the corneal astigmatic axis and the corneal astigmatism, which might be of large relevance for clinicians. For low or moderate corneal astigmatism the variation of the astigmatism axis in repeat measurements is significantly larger than for moderate or high values of corneal astigmatism. This suggests that providing one single value for the astigmatism axis uncertainty might be insufficient. Instead, we should provide the corresponding metrics for the power vector components, as the measurement uncertainty for these power vector components appears to be mostly unaffected by the amount of astigmatism. However, our study has two limitations: firstly, we used a low number of repeat measurements of 3–10. With this low number of repetitions, we cannot properly extract the distribution of the biometric measure uncertainty, meaning that we have to make assumptions about the distribution and finally recommend using a normal distribution for the measure uncertainty distribution; secondly, the study was restricted to repeat measurement data of one modern optical biometer only, and the repeat measurements were performed in one session. For other optical biometers the correlations of the biometric measures and biometric uncertainties could be different, and our inter-session repeat measurements might not be fully representative for the variation of biometric measures over a larger time interval.

In **conclusion**, our study based on sequential measurements in our study population before cataract surgery using the Haag-Streit LenStar 900 optical biometer shows excellent repeatability in measuring axial distances, and also shows that the repeatability for the corneal diameter and especially for keratometry is slightly worse. The repeatability metrics for keratometric power in the flat and steep meridian and especially for the keratometric axis are not meaningful, and instead we recommend using repeatability metrics for the power vector components, either separately for the equivalent power and the two astigmatic power vector components or for the 3D power vector components. Using the correlation data or the variance-covariance data shown in this paper a more advanced error propagation model could be implemented, to take account of potential crosstalk between the biometric measures as well as the trend of biometric measure uncertainties over the entire parameter range.
